# Multimodal interactions in insect navigation

**DOI:** 10.1007/s10071-020-01383-2

**Published:** 2020-04-22

**Authors:** Cornelia Buehlmann, Michael Mangan, Paul Graham

**Affiliations:** 1 School of Life Sciences, University of Sussex, Brighton, UK; 2 Department of Computer Science, University of Sheffield, Sheffield, UK

**Keywords:** Multimodal navigation, Cue integration, Olfaction, Vision, Insects, Ants

## Abstract

Animals travelling through the world receive input from multiple sensory modalities that could be important for the guidance of their journeys. Given the availability of a rich array of cues, from idiothetic information to input from sky compasses and visual information through to olfactory and other cues (e.g. gustatory, magnetic, anemotactic or thermal) it is no surprise to see multimodality in most aspects of navigation. In this review, we present the current knowledge of multimodal cue use during orientation and navigation in insects. Multimodal cue use is adapted to a species’ sensory ecology and shapes navigation behaviour both during the learning of environmental cues and when performing complex foraging journeys. The simultaneous use of multiple cues is beneficial because it provides redundant navigational information, and in general, multimodality increases robustness, accuracy and overall foraging success. We use examples from sensorimotor behaviours in mosquitoes and flies as well as from large scale navigation in ants, bees and insects that migrate seasonally over large distances, asking at each stage how multiple cues are combined behaviourally and what insects gain from using different modalities.

## Introduction

The world provides a host of information sources for an animal to use in controlling its behaviour, and we see in the navigation of insects the use of a variety of sensory inputs from multiple sensory modalities. Multimodal cue use allows for redundant navigation strategies and this can increase robustness, accuracy and overall foraging success. To maximise these benefits, we see that the multimodal aspects of sensory systems and navigational strategies are adapted to the insects’ specific movement patterns, lifestyle and sensory ecology. The purpose of this review is to present the current knowledge of multimodal interactions during navigation in insects. Thus, we take examples from short-range sensorimotor orientation behaviours up to large scale navigation, asking at each stage how cues are combined, what insects gain from different modalities and what we can learn about the mechanisms of these multimodal interactions.

## Multimodal orientation: lessons from mosquitoes, moths and flies

**Fig. 1:**
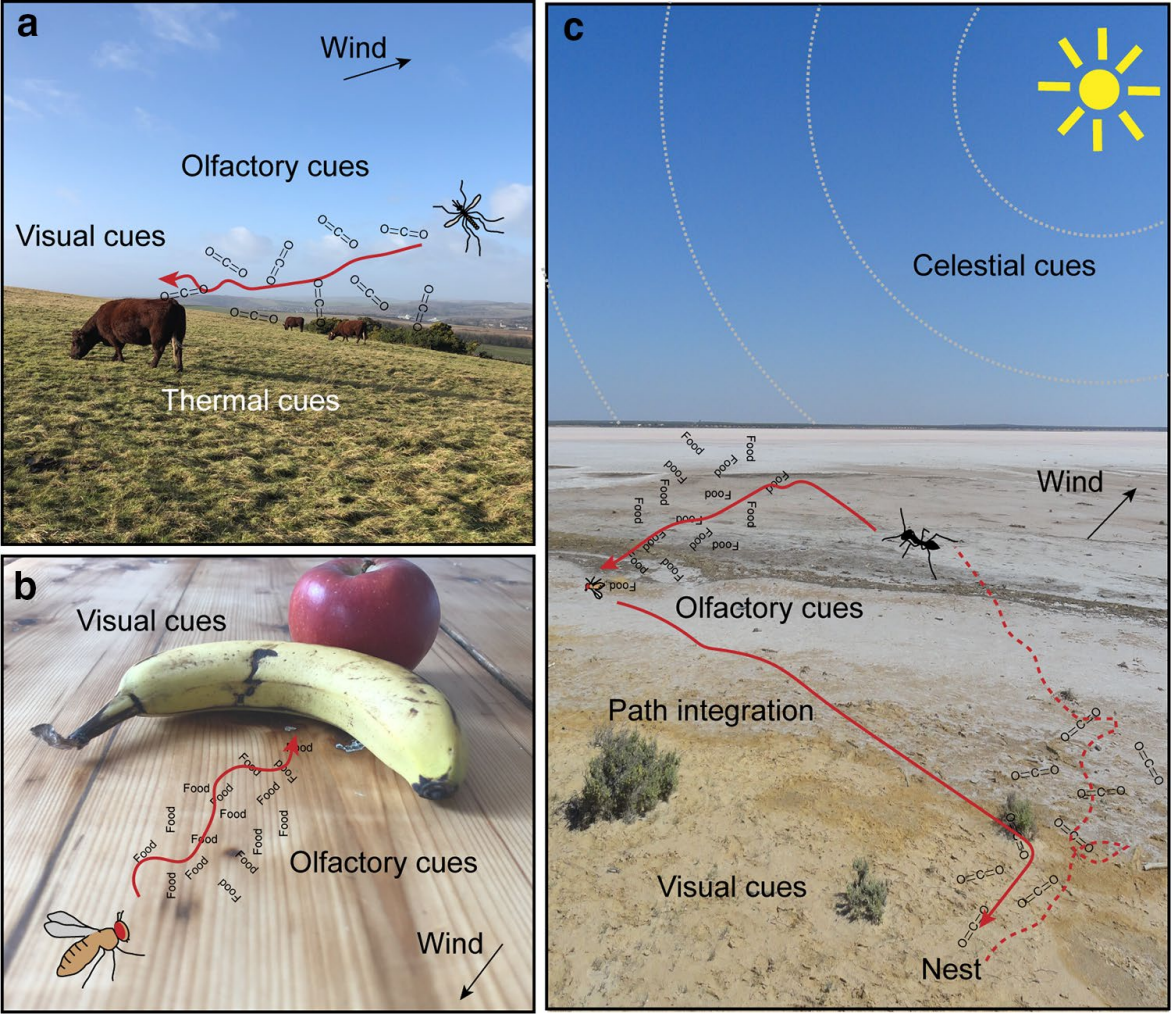
Multimodal orientation and navigation in insects. **a** Attraction to CO_2_ exhaled by a host activates a strong attraction to visual cues in mosquitoes. Once the mosquito is close to the victim, thermal cues emitted by the host are detected and used for the final approach. Here and below, the paths prior to and following sensory stimulation are shown as red dashed and red solid lines, respectively. **b** *Drosophila* flies accurately approach a piece of fruit using both olfactory and visual cues. The presence of visual cues enhances the tracking of an attractive odour plume. **c** Ants combine innate (e.g. path integration) and learnt navigational strategies to perform long and complex foraging journeys. Idiothetic information, input from sky compasses (e.g. position of the sun and the polarised pattern in the sky shown as grey dashed lines), terrestrial visual information (e.g. from vegetation), prevailing wind direction, and odours emitted by the ants’ nest, dead arthropods and the environment (odour plumes shown in black) are shown here (colour figure online)

One of the most fundamental orientation behaviours for many insects is to locate the source of an odour that may indicate food, a mating partner or oviposition site. We have all experienced how incredibly good mosquitoes are at finding us when we are enjoying a warm summer evening outdoors. When female mosquitoes need a blood meal to get proteins for their eggs, they use a combination of sensory cues to successfully localise their host. Like other insects, mosquitoes are attracted by carbon dioxide naturally exhaled by humans and other animals (Gillies 1980). Sensing CO_2_ activates a strong attraction to visual objects which allows mosquitoes to approach a host and then when in closer proximity they eventually confirm a host using thermal cues (van Breugel et al. 2015). This attraction to visual objects in the presence of CO_2_ is an elegant way for mosquitoes to be directed towards potential victims (Fig. 1a). Considering the spatial scales over which these cues can be detected, the host-seeking behaviours are often triggered sequentially, with the olfactory cues (Zollner et al. 2004) sensed from furthest away from the host followed by visual cues (Bidlingmayer and Hem 1980) and eventually thermal cues detected when very close to the target. Interestingly, the sensory modulation in mosquitoes is asymmetric with odours modulating vision but not vice versa, potentially reflecting the different effective distances of sensory modalities (Vinauger et al. 2019).

Odour-gated attraction to visual cues has also been shown in other insects such as hawk moths (Raguso and Willis 2002) and fruit flies (Fig. 1b). We all know the situation of having forgotten a delicious piece of fruit in the kitchen and ending up with a less delicious fruit and lots of fruit flies. To accurately approach such a decaying piece of fruit, flies require the use of both olfactory and visual cues. In general, it has been shown that multimodal interactions enhance performance during perception (van Swinderen and Greenspan 2003; Goyret et al. 2007; Chow and Frye 2008; van Breugel and Dickinson 2014) and learning (Rowe 2002; Guo and Guo 2005; Reinhard et al. 2006), but more specifically visual feedback is needed in flying insects for stabilizing an upwind flight (Reiser et al. 2004; Budick et al. 2007), which is a key component of plume tracking (Fadamiro et al. 1998; Frye et al. 2003). Specifically, the crossmodal interaction works because attractive odours enhance the gain of optomotor responses during flight (Chow and Frye 2008) and, therefore, through more precise flight, it is easier for the fly to track the spatial odour gradient (Duistermars and Frye 2010; Stewart et al. 2010).

So far, the highlighted studies have focused on flying insects and we don’t have the same detailed knowledge about similar cue integration in walking insects. It is known in walking cockroaches for instance, that plume-following behaviour is not enhanced in the presence of visual cues (Willis et al. 2011), however, ants do benefit from having visual information when following an odour plume, as paths are straighter with fewer turns (Buehlmann et al. 2020). Some differences may be a function of the sensory ecology being different for flying and walking insects, who encounter different challenges, even for shared orientation strategies. Volatile chemical compounds emitted by an odour source are dispersed, mixed, and diluted by air movements and form filamentous odour plumes with patchy spatiotemporal distributions of odour packets (Murlis et al. 1992, 2000; Riffell et al. 2008). However, the dynamics of olfactory information (e.g. the temporal fluctuations) in an odour plume are very different at ground level and up in the air (Fackrell and Robins 1982; Crimaldi et al. 2002).

## Multimodal navigation: lessons from ants and bees

While the challenge for some insects is to find a rotten fruit or a blood meal, some insects are capable of navigation over much larger scales, with ants and bees as the real champions. Such social insects are central place foragers and individuals become task specialists as expert navigators shuttling between their nests and foraging locations to collect food for the colony.

*Cataglyphis* desert ants are an example of one of these expert navigators, performing foraging runs of hundreds of metres when searching for sparsely distributed dead arthropods in the harsh desert environment (Wehner 1987b; Buehlmann et al. 2014; Huber and Knaden 2015). Across ants, the use of pheromone trails to recruit and navigate between the nest and reliable food locations is a common strategy (Czaczkes et al. 2015). However, in addition to, or even instead of trail pheromones, many ant species can navigate using personal navigational strategies (Wehner et al. 1996; Wehner 2003, 2008; Collett et al. 2013; Knaden and Graham 2016). In this review, we focus on such individually navigating ants whose recipe for navigational success is the clever combination of innate navigational strategies and the learning of key features from the environment (Fig. 1c; Wehner 2003, 2008; Collett et al. 2013; Knaden and Graham 2016).

One widespread innate navigational strategy is path integration (PI), where ants keep track of direction (Wehner and Mueller 2006) and distance travelled (Wittlinger et al. 2006) and continually integrate this information such that they can travel directly to their nest from any point during a foraging journey (Wehner and Srinivasan 2003; Ronacher 2008; Collett 2019). This innate strategy allows ants to explore the environment while being safely connected to the nest and, furthermore, gives foragers the chance to learn relevant environmental cues about locations and foraging routes. Such learnt information about routes (olfactory cues: (Buehlmann et al. 2015; Huber and Knaden 2017), visual cues: (Collett et al. 1992; Baader 1996; Kohler and Wehner 2005; Macquart et al. 2006; Mangan and Webb 2012)) and important places (olfactory cues: (Steck et al. 2009), visual cues: (Wehner and Raeber 1979; Wehner et al. 1996; Knaden and Wehner 2005), tactile cues: (Seidl and Wehner 2006), magnetic cues: (Buehlmann et al. 2012a), vibration cues: (Buehlmann et al. 2012a), thermal cues: (Kleineidam et al. 2007)) can come from a range of sensory modalities and is essential for successful and accurate navigation.

Bees are fascinating navigators too, travelling up to several kilometres when visiting flower patches (Janzen 1971; Osborne et al. 2008; Pahl et al. 2011; Woodgate et al. 2016b). Within these foraging patches, we see multimodality for the successful detection of flowers, with bees using a combination of floral cues, such as temperature (Harrap et al. 2017), tactile cues (Kevan and Lane 1985), odours (Bhagavan and Smith 1997), floral iridescence (Whitney et al. 2009), flower pattern (de Ibarra and Vorobyev 2009) or colours (Lawson et al. 2017). Loaded with pollen or nectar from the flower, bee foragers then navigate back to their hive using a combination of path integration (Srinivasan 2015) and learnt terrestrial visual cues (see e.g. (Towne et al. 2017; Menzel et al. 2019)).

In the following section, we highlight interesting features of multimodality within and between the navigational strategies highlighted above. We hope to demonstrate themes within insect navigation mechanisms that show how multimodality helps animals adapt to their specific habitat and increase robustness to complex dynamic environments.

## Multimodality within the insect compass system

Idiothetic information alone cannot guide animals over long distances (Cheung et al. 2007), as shown by the curved paths of humans trying to maintain a straight course in featureless environments (Souman et al. 2009) or the failure of wandering spiders to accurately return to a safe place when external orientation cues are removed (Seyfarth et al. 1982). To compensate for such errors, insects have evolved an array of compass systems that allow for more precise orientation, as most beautifully demonstrated by the yearly migrations of millions of monarch butterflies from their summer feeding grounds in southern Canada to roosting sites in central Mexico (Brower 1995) relying on a highly tuned sense of direction (Reppert et al. 2010).

**Fig. 2:**
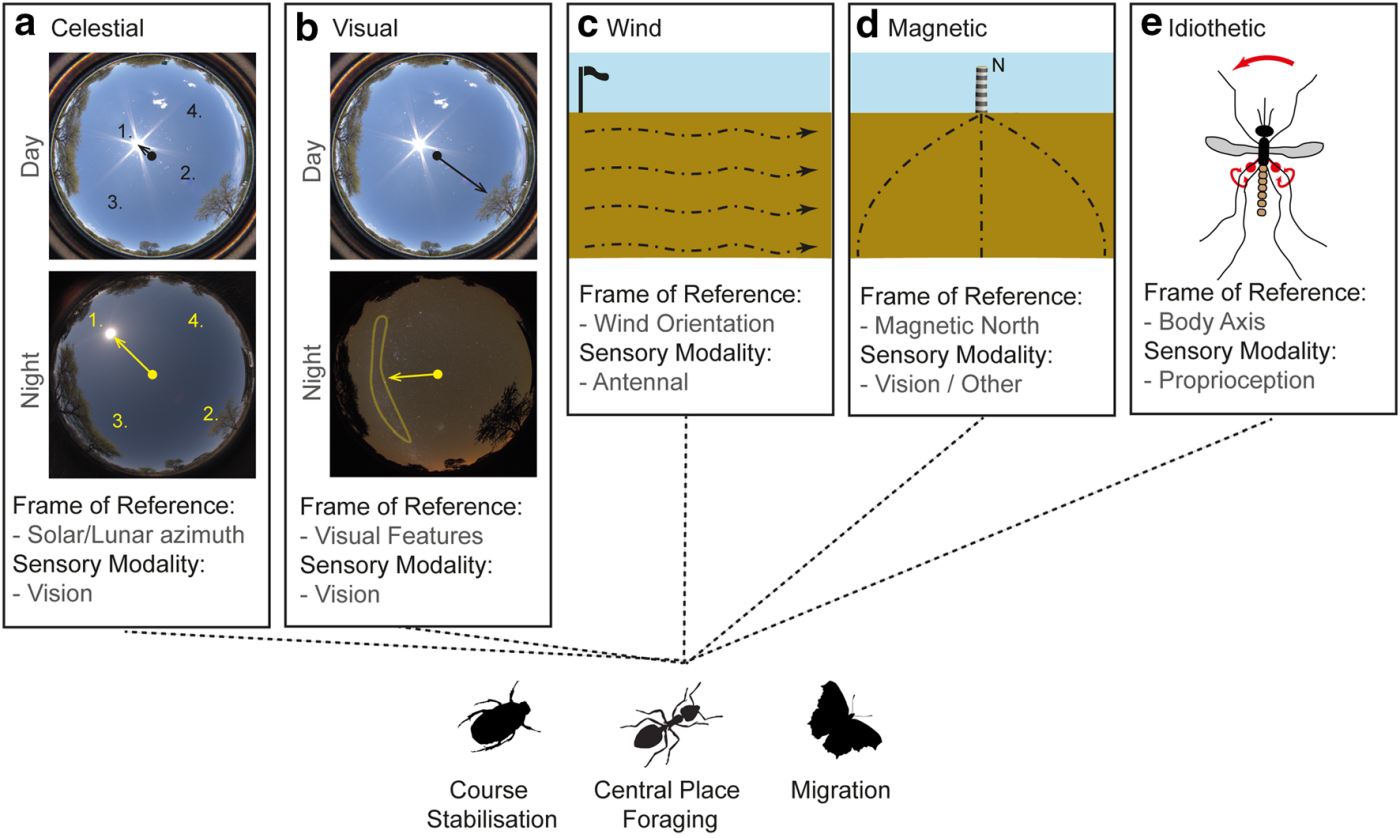
Multimodal compasses in insects. **a** Insects derive their orientation with respect to the solar and lunar azimuths either through direct observation (1.) or indirect measures (2. detection of the contrasolar/contralunar azimuth which has the highest degree of polarisation; 3. measurement of chromatic gradients; 4. sampling the polarised light pattern). **b** Insects also orient with respect to prominent visual features such as the visual panorama (top) or the milky way (bottom). Background fisheye images are sampled from dung beetle habitats in South Africa and provided by Dr James Foster. **c** Consistent wind provides a short-term orientation cue known to be used by ants and dung beetles. **d** Insects derive their orientation with respect to the Earth’s North–South axis but the sensory pathways remain unresolved. **e** Proprioception tracks changes in animal heading iteratively, which is suitable for direction tracking over short durations, for instance, changing direction alters haltere orbits. Insect species combine these multimodal and multiscale compasses differently depending on their navigational need, for example, simple course stabilisation in dung-beetles, central-place navigation in ants, and long-range migration in butterflies

Across insects, the most well-studied compass system is the highly conserved celestial compass (Fig. 2a; ants: (Wehner 1984; Collett and Collett 2000); bees: (Hardie 1986); wasps: (Ugolini 1987); flies: (Weir and Dickinson 2012; Giraldo et al. 2018); desert locusts: (Mappes and Homberg 2004); monarch butterflies (Merlin et al. 2012); dung beetles: (el Jundi et al. 2019)) by which animals track their orientation with respect to the solar or lunar azimuth, through both direct visual detection of the light source (sun compass: (Wehner and Mueller 2006; Beetz and el Jundi 2018; Santschi 1911); moon compass (Dacke et al. 2004)), or by inference of its position through observation of correlated chromatic intensity gradients (diurnal: (Pfeiffer and Homberg 2007; el Jundi et al. 2014)), and polarised light patterns in the sky (diurnal: (Wehner 1976; Wehner and Labhart 2006; Wehner and Mueller 2006); nocturnal: (Wehner and Duelli 1971; Dacke et al. 2003a, b)). Theoretically, the insect visual system could derive a celestial compass bearing accurate to less than one degree (Gkanias et al. 2019) but as the cues constantly move with respect to their observer, due to the rotation of the earth, compensation mechanisms are required for long-term use (see e.g. (Wehner and Lanfranconi 1981; Dyer 1987; Towne and Moscrip 2008)).

Insects can also derive local short-term compasses by tracking their orientation relative to visual features in their surroundings (Fig. 2b; Lent et al. 2010; Seelig and Jayaraman 2015; Buehlmann et al. 2016; Varga and Ritzmann 2016; Woodgate et al. 2016a)) and the prevalent wind direction (Fig. 2c; Wehner and Duelli 1971; Bell and Kramer 1979; Heinzel and Bohm 1989; Wolf and Wehner 2000; Mueller and Wehner 2007; Chapman et al. 2008; Dacke et al. 2019)) most likely detected through the Johnson’s organ in the antennae (Wehner and Duelli 1971; Gewecke 1974; Dacke et al. 2019).

Furthermore, insects can also track their orientation with respect to the Earth’s magnetic field (Fig. 2d; Collett and Baron 1994; Guerra et al. 2014; Dreyer et al. 2018; Fleischmann et al. 2018)) which is largely stationary, but is likely much less accurate than a celestial compass (Mouritsen 2018). Insects sense their bearing with respect to the magnetic North–South axis either through trophocyte cells containing super-paramagentic magnetite that change activity relative to an induced magnetic field (Hsu and Li 1994; Hsu et al. 2007), and/or crypotchrome activity in the visual pathways (Gegear et al. 2008; Phillips et al. 2010; Reppert et al. 2010).

Finally, despite its inherent susceptibility to accumulative errors, insects can also track their orientation using proprioceptive cues derived from leg joint angles, and/or the halteres (Fig. 2e; Wehner 1992; Kim and Dickinson 2017)).

Taken as a whole, the evidence above shows the exploitation of a vast range of sensory cues. Insects are able to combine these multiscale, multireference, and multimodal compasses in different, flexible manners depending on the context or particular ecology. For example, dung beetles trying to maintain a straight course away from the crowded dung pile, simply minimise any change in sensory input across their short journey (el Jundi et al. 2016, 2019; Dacke et al. 2019), whereas central place foragers that visit the same feeding site over successive days, or migratory insects that navigate for long periods per day, use matched filters (Wehner 1987a, 1989; Bech et al. 2014; Warrant 2016) to derive a robust, time-invariant, geocentric compass. Desert ants and dung beetles demonstrate the flexible transfer of orientation information from one modality to another (wind to celestial: (Wystrach and Schwarz 2013; Dacke et al. 2019); visual to celestial (Schwarz et al. 2017a); between polarisation and sun compass (Lebhardt and Ronacher 2015)) to markedly extend their behavioural capacity. Moreover, multiple celestial cues can be used simultaneously and when cues are experimentally set in conflict, the insects’ headings often represent an intermediate direction with different cues weighted depending on the relative strength of their input (Lebhardt and Ronacher 2014; Wystrach et al. 2014).

## How sensory ecology drives the balance of cue use

One way of looking at how cues from different modalities interact with each other is to look at how different species have adapted to their particular habitat or sensory ecology. We have seen in the previous section that dung beetles can use multiple celestial cues such as the sun, the moon and the pattern of polarised light for their straight-line orientation away from the dung pile. Interestingly, when we look at celestial cue use in diurnal and nocturnal beetles experiencing different light levels, we find the same orientation behaviours in both species, but we see differences in the cue weighting (el Jundi et al. 2015), which dynamically allows beetles to successfully orient across different environmental conditions using whatever compass cue is available and reliable.

In individually foraging desert ants that don’t use pheromone trails, there are interesting interactions between path integration and visual guidance that vary with habitat. Closely related desert ant species inhabit similar ecological niches as they are all thermophilic scavengers, but their environments can differ fundamentally, with different levels of clutter and vegetation, thus, different amounts of visual information available for navigation. For instance, North African *Cataglyphis fortis* foragers navigate through sparsely vegetated salt pans while Australian *Melophorus bagoti* ants inhabit a densely cluttered habitat. We know from both ant species that they use the visual panorama for navigation (*C. fortis*: (Huber and Knaden 2015); *M. bagoti*: (Graham and Cheng 2009a, b)). But unsurprisingly, we see that *C. fortis* ants inhabiting an environment that is poor in visual information rely strongly on path integration, while *M. bagoti* ants rely more strongly on terrestrial visual cues and show less trust in their PI vector (Buehlmann et al. 2011). Looking across multiple species, we see a general trend that ants were taken from a feeder and released in an unfamiliar location follow path integration before starting a systematic nest search (Wehner and Srinivasan 1981; Schultheiss and Cheng 2011), with the proportion of the home vector followed being inversely proportional to the typical density of vegetation (*Cataglyphis fortis*: (Buehlmann et al. 2011); *Melophorus bagoti*: (Narendra 2007b; Buehlmann et al. 2011; Cheng et al. 2012; Schultheiss et al. 2016); *Formica japonica*: (Fukushi 2001; Fukushi and Wehner 2004); *Gigantiops destructor*: (Beugnon et al. 2005)). These results indicate that the weighting of different navigational strategies differs across species, with ants from visually rich habitats relying less heavily on PI (see also (Cheung et al. 2012)). Moreover, these species-specific behavioural differences are further shaped by the ants’ very specific local habitat characteristics. Experiments performed with *M. bagoti* ants reveal that ants from a nest in a highly cluttered area relied less strongly on PI when displaced to an unfamiliar test field than ants from a more open area (Cheng et al. 2012). Hence, a crucial factor in these displacement tests is the visual mismatch between the ants’ memorised views and the views experienced at the novel location (see also (Islam et al. 2020)). Moreover, experienced ants with a strong memory of learnt visual scenes along their habitual foraging route will experience a higher visual mismatch when displaced to a novel location than naïve ants, and thus run off a shorter proportion of their home vector when displaced (Schwarz et al. 2017b).

**Fig. 3:**
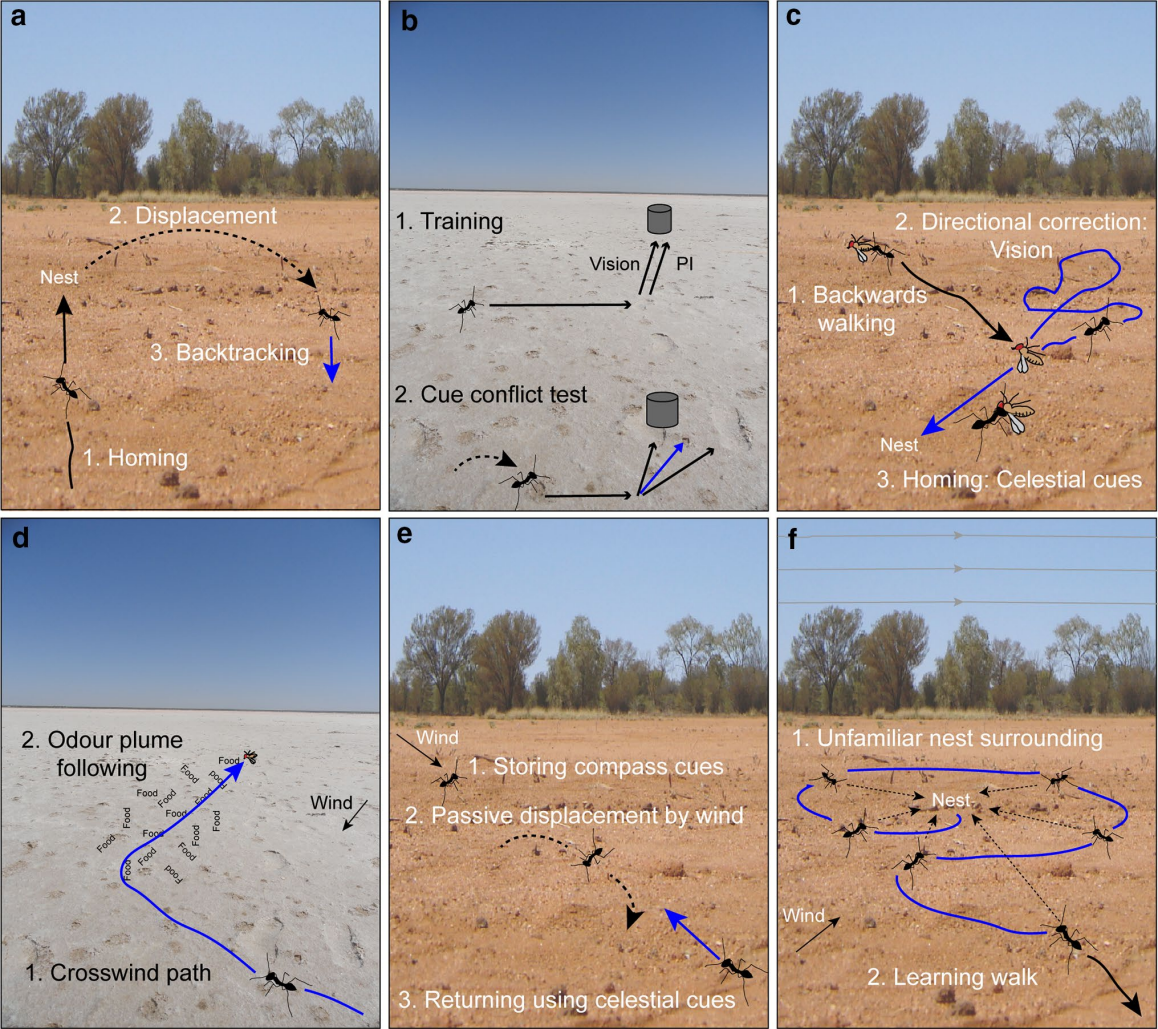
Examples of multimodal interactions in navigating ants. **a** If an ant runs off her entire PI vector (1.), is captured near her nest entrance and displaced and released at a novel location (2.), she will search for the nest. Ants from visually rich environments do not show random initial directions, rather, their bearings are biased in the nest-to-feeder direction (3.). The recent experience of the visual surroundings near the nest leads to this backtracking behaviour. **b** Ants are trained to navigate along a route (shown as arrows) using PI and visual guidance (1.). PI and visual cues are used simultaneously and when information from PI and visual guidance are in conflict (2.), ants head in intermediate directions (shown in blue). **c** Ants with a large piece of food walk backwards (1.). Occasionally they drop their piece of food and perform small loops, allowing familiar visual scenes to correct their heading direction (2.). Backwards walking is resumed with their updated heading subsequently controlled by celestial compass information (3.). **d** Foraging desert ants combine a high sensitivity to food odours with specific movement patterns that increase the time they spend moving in crosswind paths and this combination of wind (1.) and odour information (2.) increases food detection speeds. **e** When there are strong gusts of wind, ants ‘clutch’ to the ground to resist being blown away, and compute and store the compass direction of the wind using their current heading and the relative direction of the wind to their body (1.) After they are blown away (2.), ants can use the integrated information to walk back in the direction opposite to the one they had just been blown away (3.). **f** At the beginning of each ants’ foraging career or when the appearance of the world has changed (1.), ants start with short excursions in the close vicinity of the nest (2.). The characteristically structured elements of these learning walks are shaped by multiple information sources, such as PI, terrestrial visual information, wind direction, or the earth’s magnetic field (shown as grey lines in the sky) (colour figure online)

Above we have seen how interactions between path integration and visual guidance are adaptive and tailored to a species’ sensory ecology. We see similar interactions in systematic nest-search behaviours. If an ant runs off its entire PI vector, is captured near its nest entrance and then released at a novel location, it will search for the nest (Wehner and Srinivasan 1981; Schultheiss and Cheng 2011). Upon release, ants from featureless environments will initially head in a random direction before producing a search distribution that is symmetrical about the release point (Wehner and Srinivasan 1981). Ants that are experienced in visually rich environments do not show these random initial directions when displaced, rather, their bearings are biased in the nest-to-feeder direction (Wystrach et al. 2013), i.e. the ants walk opposite to the direction they had just travelled (Fig. 3a). Experiments show that it is the recent experience of the visual surroundings near the nest that leads to this backtracking behaviour (Wystrach et al. 2013; Graham and Mangan 2015). In summary, we see adaptive interactions between navigational strategies and the nature of these interactions depends on the animals’ sensory ecology as well as individual experience.

## How insects apply multimodal information

Ants can benefit from the use of multiple navigational cues (see above) because in some situations cues act additively in determining the ants’ path accuracy (Buehlmann et al. 2020). However, in addition to a general improvement in accuracy, there are nice examples of more complex interactions in how multiple cues interact to control spatial behaviour.

The egocentric nature of path integration means that small errors accumulate along a path (Sommer and Wehner 2004; Merkle et al. 2006). This is why it is so important to supplement PI with the learning and use of other cues (Knaden and Graham 2016). Thus, at most times in a forager’s life an ant will have PI, as well as learnt environmental information, available for guidance between the nest and a foraging site. The integration of path integration and vision is relatively well studied, and behavioural experiments have shown that these cues are used simultaneously, i.e. they are redundant navigational strategies that contribute to an ant’s heading direction during its journey (Narendra 2007a, b; Reid et al. 2011; Collett 2012; Legge et al. 2014) and might even be weighted optimally based on their reliability (Vickerstaff and Cheung 2010; Legge et al. 2014; Wystrach et al. 2015). A typical way of looking at such interactions between PI and visual guidance is to observe the ants’ behaviour when the direction indicated by the path integration system is at odds with the information from visual cues. In experiments, creating a subtle conflict between the PI vector and the direction indicated by the familiar visual scene usually results in ants heading in intermediate, compromise directions (Fig. 3b; Collett 2012; Wehner et al. 2016)).

Another example of how visual guidance interacts with other guidance systems comes from ants walking backwards which they do when they have to move a large piece of food (Fig. 3c). When moving backwards, ants are able to approach their nest either under the control of PI (Pfeffer and Wittlinger 2016) or without PI information (Ardin et al. 2016), the latter case suggesting guidance by familiar visual cues. One notable feature of ants’ paths when moving backwards is that they occasionally drop their piece of food and perform small loops nearby (Pfeffer and Wittlinger 2016). During these loops, familiar visual scenes may be experienced that allow the ants to set an accurate direction for the route once they reacquire the food and resume their journey, albeit with their heading being controlled by celestial compass information, not the familiar visual scene, whilst moving backwards (Schwarz et al. 2017a).

We have these interesting examples of how PI and visual guidance influence the behaviour of navigating ants, but we can also ask about the role of olfaction in individually navigating ants and how odour use interacts with other navigational strategies. Following an attractive odour plume up to its source (Fig. 1) is a common strategy seen in many insects such as flies (Budick and Dickinson 2006; van Breugel and Dickinson 2014), moths (Baker and Kuenen 1982; David et al. 1983; Kennedy 1983; Carde and Willis 2008; Willis et al. 2013), cockroaches (Willis and Avondet 2005) or ants (Wolf and Wehner 2000; Buehlmann et al. 2012b, 2014). Desert ants, when searching for perished arthropods, combine a high sensitivity to food odours with specific movement patterns that increase the time they spend moving crosswind (Buehlmann et al. 2014). This combination of wind and odour information use increases food detection speeds in the harsh Tunisian salt pan (Fig. 3d). After discovering a food item, the next challenge is to safely return back to the nest where olfactory information can also be useful (Steck et al. 2009). Homing *C. fortis* follow the PI vector back to the close vicinity of the nest from where they pinpoint it by walking upwind, i.e. they follow a nest-derived CO_2_ plume back to the nest (Buehlmann et al. 2012b). In homing ants, PI overrides olfactory information and ants only respond to nest odours when they are close to home, which is crucial, as homing ants may pass through multiple CO_2_ plumes emanating from foreign nests at earlier stages of their homeward journey (Buehlmann et al. 2012b). However, in the same species, foraging ants heading to a regular foraging area always find food odours attractive and thus olfactory information overrides PI information about feeder locations (Buehlmann et al. 2013). This is a clever way of avoiding entering a wrong nest but maximising foraging efficiency.

We have seen before that compass information can be transferred from one modality to another (e.g. (Dacke et al. 2019)). In ants, we see additional interactions between the visual compass and wind information when they are blown away from their familiar route by a sudden gust (Fig. 3e). The moment before they are blown away, ants ‘clutch’ on the ground to resist the wind and this is the moment they compute and store the compass direction of the wind using their current heading and the relative direction of the wind to their body (Wystrach and Schwarz 2013). If their clutching behaviour fails, and they are blown away, they can use the integrated information to walk back in the direction opposite to the one they had just been displaced (Wystrach and Schwarz 2013).

## How innate behaviours support learning

In the examples of orientation and navigation above we have shown how multimodal interactions shape behaviour. Such interactions are also present during the learning of environmental cues. The topic of insect learning, in general, is discussed elsewhere (e.g. reviewed in (Avargues-Weber et al. 2011; Giurfa 2013; Perry et al. 2017)) and we focus in this section on how innate behaviours shape learning for navigation. At the beginning of each forager’s career or when the appearance of the world has changed, individuals do not immediately perform long foraging journeys but implement little excursions in the close vicinity of the nest and these choreographed movements allow the learning of key features from the environment (Collett and Zeil 2018; Zeil and Fleischmann 2019). These so-called learning walks or flights are essential to learn information required on subsequent foraging journeys (ants: (Judd and Collett 1998; Nicholson et al. 1999; Wehner et al. 2004; Graham and Collett 2006; Mueller and Wehner 2010; Stieb et al. 2012; Fleischmann et al. 2016, 2017; Grob et al. 2017; Jayatilaka et al*.* 2018), bees: (Philippides et al. 2013; Degen et al. 2015), wasps: (Collett and Lehrer 1993; Stuerzl et al. 2016)). The characteristically structured elements of these paths are matched to the ants’ visual ecology (Fleischmann et al. 2017) and are also shaped by multiple information sources (Fig. 3f), such as PI (Graham et al. 2010; Mueller and Wehner 2010), wind direction (Vega Vermehren et al. 2020) or the Earth’s magnetic field (Fleischmann et al. 2018).

Innate navigational strategies such as path integration, pheromone trails and innate responses to ecologically relevant stimuli can also all facilitate learning (Voss 1967; Collett 1998, 2010; Heusser and Wehner 2002; Collett et al. 2003; Graham et al. 2003, 2010; Mueller and Wehner 2010; Graham and Wystrach 2016). PI safely connects an ant with its nest during a learning walk, but it is also essential for longer distance scaffolding. For instance, ants using PI in unfamiliar terrain will take consistent and direct paths, potentially simplifying the learning of visual information along a route (Collett et al. 2003). Importantly, although PI plays an important role in the learning of visual information, PI itself involves little or no learning (Narendra et al. 2007; Merkle and Wehner 2009). Moreover, the visual cues can later be retrieved and utilised independently of the path integration system (Collett et al. 1992, 2001; Kohler and Wehner 2005; Mangan and Webb 2012). Another interaction between PI and visual learning is the modulation of visual experience via walking speed (Buehlmann et al. 2018) where the ants’ speed is modulated in a way that might help ants search for, utilise or learn environmental information at important locations. Therefore, path integration mediated movement characteristics might assist ants in adequately learning or responding to sensory cues at locations of importance, by allowing those other cues to act for a longer period of time (see also (Chittka et al. 2009)).

Learning is facilitated by innate strategies but there are also synergies between sensory modalities when different cue types are being learnt. Ants learning bimodal cues (visual and olfactory cues presented together) learn faster than ants that only have single cues to learn (Steck et al. 2011). Interestingly, we see that bimodal landmarks are first learnt as their individual components but later stored as a unit. That means, although initially the presence of a second sensory cue enhances learning performance of a unimodal cue (Steck et al. 2011), the components of the bimodal cue are often fused together after several training trials and ants will no longer respond accurately to either of the components presented alone (Steck et al. 2011; Buehlmann et al. 2020). Although this cue binding can be shown to be dynamic and depends both on the navigational context and the specific information provided by each modality (Buehlmann et al. 2020).

## Conclusions

In this review, we have shown the rich and diverse ways that evolution has provided insects with mechanisms for orientation and navigation using multimodal information. We have seen that multimodal strategies and information sources interact in many ways and navigational strategies are tuned to the current needs of behaviour and the specific sensory ecology. This further confirms that insects, despite their small brains, are sophisticated and dynamic in their spatial cognition and not in the least simple or robotic.
